# Announcement: 8th International Symposium on Platinum and Other Metal Coordination Compounds in Cancer Chemotherapy ISPCC '99

**DOI:** 10.1155/MBD.1998.319

**Published:** 1998


					8TH INTERNATIONAL SYMPOSIUM
ON PLATINUM AND OTHER METAL
COORDINATION COMPOUNDS
IN CANCER CHEMOTHERAPY
ISPCC '99
March 28-31 1999, Oxford, UK
Conference Directors
L. R. Kelland
Local Organising Committee
L. Robertson
F. Raynaud
Scientific Committee
M. Abrams
R. Brown
E. Cvitkovic
A. Eastman
L. C. Erickson
T. Hambley
S. B. Howell
J. S. Lazo
G. Los
F. Muggia
J. Reedijk
J. H. M. Schellens
J H. Schornnagel
E. G. E. de Vries
General Information
I. R. Judson
S. Y. Sharp
P. Workman
P. A. Andrews
H. Calvert
J. C. Chottard
M. Egorin
N. Farrell
T. Hamilton
Y. Kidani
S J Lippard
M. J McKeage
H. M. Pinedo
P. Sadler
Z. H. Siddik
W. J. F. van der Vijgh
R. Wood
All activities will be hosted by Keble College in the centre of the beautiful historic city of Oxford and
within easy reach of Heathrow and Gatwick airports.
If you are interested in attending this Symposium, please contact the organisers.
Contact address
Dr Lloyd R. Kelland
CRC Centre for Cancer Therapeutics
The Institute of Cancer Research
15 Cotswold Road, Sutton SM2 5NG
Surrey, UK
Tel: (44) (0)181 643 8901
Email: lloyd @ icr.ac.uk
You will then receive the second announcement with further details about the program, registration
and abstract forms.
The registration fee (approximately 325 for early registration pre-lst December 1998) will cover all
sessions, accommodation and meals.
Each session will begin with 1-2 keynote review(s), followed by proffered oral and poster
presentations.
319
Monday, March 29th 1999
am 1. SYNTHESIS AND ACTIVITY OF NEW COMPOUNDS
Convenors:
Abrams M Langley Farrell N Richmond
Harnbley T Sydney Kidani Y- Fujisawa
To include: "Preclinical new platinum drugs, other metals"
pm 2. PRECLINICAL AND CLINICAL PHARMACOLOGY
Convenors:
Egorin M Baltimore McKeage M Auckland
Raynaud F Sutton Schellens J Amsterdam
Siddik Z Houston van der Vijgh W Amsterdam
To include: "Preclinical interactions with other drugs, delivery applications, toxicology, nuclear
imaging"
Tuesday, March 30th 1999
am 3. PLATINUM DRUG RESISTANCE
Convenors:
Andrews P- Rockville
Erickson L- Indianapolis
Los G La Jolla
To include: "Genomics and platinum drugs; novel resistance mechanisms"
de Vries E Groningen
Hamilton T- Philadelphia
Sharp S Sutton
pm 4. DRUG DNA/PROTEIN INTERACTIONS
Convenors:
Chottard J Paris Sadler P Edinburgh
Lippard S Cambridge Wood R South Mimms
Reedijk J Leiden
To include: "DNA repair pathways, HMG proteins, DNA protein kinases, telomerase"
Wednesday, March 31 st 1999
am 5. CLINICAL TRIALS (sponsored by ZENECA Pharmaceuticals)
Convenors:
Calvert A Newcastle Cvitkovic E Villejuif
Judson Sutton Muggia F New York
Pinedo H Amsterdam Schornagel J Amsterdam
To include: "How effective are the new platinum drugs? Oxaliplatin, JM216, SPI-077, ZD0473,
BBR3464; cisplatin/radiation/new drugs trials"
pm 6. MOLECULAR PHARMACOLOGY/APOPTOSIS
Convenors:
Brown R Glasgow Eastman A- Hanover
Howell S La Jolla Kelland L Sutton
Lazo J Pittsburgh Workman P Sutton
To include: "How does platinum kill cells? Interaction with signal transduction/cell death pathways"
320

				

